# Serum neurotransmitter imbalances in benign paroxysmal positional vertigo: correlations with anxiety, depression, and sleep quality

**DOI:** 10.3389/fneur.2026.1798705

**Published:** 2026-04-10

**Authors:** Qian Zhang, Ruifang She, Haiyan Zhou, Jinying Guo, Zunjie Hu

**Affiliations:** 1 Department of Neurology, The Affiliated Tai’an City Central Hospital of Qingdao University, Tai’an, Shandong, China; 2 Department of Chinese Medicine, The Affiliated Tai’an City Central Hospital of Qingdao University, Tai’an, Shandong, China; 3 Department of Endocrine, The Affiliated Tai’an City Central Hospital of Qingdao University, Tai’an, Shandong, China; 4 Department of Urology, The Affiliated Tai’an City Central Hospital of Qingdao University, Tai’an, Shandong, China

**Keywords:** anxiety, benign paroxysmal positional vertigo (BPPV), correlation analysis, dopamine, epinephrine, norepinephrine, sleep quality

## Abstract

**Purpose:**

This study aims to investigate the associations between serum neurotransmitter levels (dopamine, epinephrine, norepinephrine) and psychological/sleep comorbidities in patients with benign paroxysmal positional vertigo (BPPV), elucidating the neurobiological mechanisms underlying their interplay.

**Methods:**

A prospective cross-sectional study enrolled 310 BPPV patients and 300 age- and gender-matched healthy controls. Psychological status was assessed using the Hamilton Anxiety Rating Scale (HAMA), Hamilton Depression Rating Scale (HAMD), and Pittsburgh Sleep Quality Index (PSQI). Serum neurotransmitters were quantified via high-performance liquid chromatography–tandem mass spectrometry (HPLC-MS/MS). Spearman’s correlation analysis evaluated relationships between neurotransmitters, psychological/sleep scores, and functional disability (Dizziness Handicap Inventory, DHI).

**Results:**

Benign paroxysmal positional vertigo patients exhibited significantly higher HAMA, HAMD, and PSQI scores compared to controls (all *p* < 0.0001). Serum dopamine (*p* = 0.0048), epinephrine (*p* < 0.0001), and norepinephrine levels (*p* < 0.0001) were significantly decreased in BPPV patients. Correlation analysis revealed that lower epinephrine and norepinephrine levels were strongly associated with greater physical impairment (*r* = −0.658 and −0.694, respectively, both *p* < 0.0001) and more severe depressive symptoms (epinephrine vs. HAMD: *r* = −0.704, *p* < 0.0001). A strong positive correlation existed between DHI and HAMA (*r* = 0.701, *p* < 0.0001), indicating shared influences on functional disability and anxiety.

**Conclusion:**

Benign paroxysmal positional vertigo is associated with monoaminergic neurotransmitter deficits, which correlate with heightened anxiety, depression, and physical impairment. These findings highlight neurotransmitter imbalances as key mediators of the bidirectional relationship between vestibular dysfunction and psychological disturbances, supporting the need for integrated assessments of neurochemical and emotional parameters in BPPV management.

## Introduction

Benign paroxysmal positional vertigo (BPPV), the most prevalent peripheral vestibular disorder, manifests as transient vertigo episodes triggered by gravity-dependent head movements, typically lasting < 1 min (1, 2). Mechanistically, dislodged otoconia—calcium carbonate crystals—migrate into semicircular canals, most commonly the posterior canal (59–90% of cases), inducing aberrant endolymph dynamics and vestibular signaling (3, 4). With a lifetime prevalence of 2.4% and annual recurrence rates of 15–20%, BPPV imposes substantial socioeconomic burdens (5, 6). Diagnosis relies on positional maneuvers (e.g., Dix-Hallpike, supine roll tests) to elicit pathognomonic nystagmus patterns, as outlined by the Bárány Society criteria (7). While these maneuvers remain the gold standard, emerging objective tests, such as video head impulse test and vestibular evoked myogenic potentials, are being explored for their diagnostic utility in BPPV, although their routine clinical applicability is still under investigation (8). First-line treatments, such as canalith repositioning procedures (CRPs), demonstrate immediate efficacy (6). However, challenges persist, including recurrent or refractory cases, canal conversions, and post-treatment complications (6). Etiologically, BPPV remains idiopathic in 85% of cases, though associations with trauma, osteoporosis, hypertension, and vitamin D deficiency highlight multifactorial pathways (9–11). However, molecular mechanisms underlying BPPV remain partial, necessitating translational research to identify biomarkers and targeted therapies.

Beyond the physical manifestations, BPPV is closely intertwined with psychological and sleep-related disturbances that significantly impact patients’ quality of life. Studies have consistently reported a higher prevalence of anxiety and depression in BPPV populations, with one meta-analysis indicating that 27% of BPPV patients experience anxiety, representing a 3.19-fold increased risk compared to healthy controls (12). Furthermore, the severity of vertigo symptoms has been shown to correlate significantly with the degree of anxiety and depression (13), underscoring the close link between physical and psychological distress. Meanwhile, these psychological symptoms are often accompanied by poor sleep quality (14, 15). For example, a cross-sectional study demonstrated that BPPV patients exhibit significantly higher Pittsburgh Sleep Quality Index (PSQI) scores, reflecting compromised sleep efficiency, increased wakefulness after sleep onset, and reduced deep sleep duration (16). The interplay between BPPV and these factors is bidirectional: recurrent vertigo episodes disrupt sleep architecture, while sleep disorders exacerbate vestibular dysfunction and emotional distress (14). Psychological distress in BPPV, measured by tools such as the Hamilton Anxiety Rating Scale (HAMA) and Hamilton Depression Rating Scale (HAMD), correlates with functional disability as assessed by the Dizziness Handicap Inventory (DHI) (4, 17, 18). Notably, female patients and those with longer disease durations show more pronounced sleep disturbances, suggesting sex- and duration-dependent vulnerability (19). Despite these associations, the underlying molecular mechanisms linking BPPV to anxiety, depression, and sleep disorders remain unclear. Proposed pathways include dysregulation of the hypothalamic–pituitary–adrenal axis, neurotransmitter imbalances (e.g., catecholamines), and central vestibular-cortical interactions (14, 20), but empirical evidence for these mechanisms is limited.

Given the clinical relevance of these psychological and sleep comorbidities, there is a critical need to investigate their biological underpinnings in BPPV. This study integrated psychological assessments (HAMA, HAMD, and PSQI) with high-precision liquid chromatography–tandem mass spectrometry (HPLC-MS/MS) to systematically analyze the correlations between serum neurotransmitter levels, psychological/sleep scores, and dizziness-related disability in BPPV patients. The aim was to explore the role of monoaminergic pathways in the interplay between vestibular dysfunction, emotional distress, and sleep disturbances. This research not only identifies potential molecular biomarkers for BPPV comorbidities but also highlights the importance of neurotransmitter regulation as a multidimensional therapeutic target.

## Materials and methods

### Study design and participants

This prospective cross-sectional study enrolled participants from January 2024 to December 2024. Data were prospectively collected during standardized assessments at the outpatient clinic of Taian Central Hospital. A total of 310 patients diagnosed with benign paroxysmal positional vertigo (BPPV) and 300 age- and sex-matched controls were enrolled. The study protocol was approved by the Institutional Ethics Committee of Taian Central Hospital (Approval No.: 2023-05-09), and written informed consent was obtained from all participants.

The BPPV was diagnosed according to the 2017 guidelines of the Chinese Society of Otorhinolaryngology-Head and Neck Surgery. Inclusion criteria included: (1) Recurrent, transient (≤1 min) vertigo triggered by changes in head position relative to gravity; (2) Positional nystagmus confirmed via the Dix-Hallpike or supine roll test. Exclusion criteria encompassed other vestibular disorders (e.g., Ménière’s disease, vestibular migraine, vestibular neuritis, central positional vertigo), systemic illnesses affecting balance (e.g., orthostatic hypotension, posterior circulation ischemia) and a pre-existing, physician-diagnosed major psychiatric disorder (e.g., major depressive disorder, generalized anxiety disorder) prior to the onset of BPPV symptoms. Healthy controls were rigorously screened to exclude individuals with: (1) Vestibular disorders (e.g., Ménière’s disease, vestibular migraine, vestibular neuritis, central positional vertigo); (2) Psychiatric conditions (e.g., anxiety, depression); and (3) Systemic illnesses affecting balance (e.g., orthostatic hypotension, posterior circulation ischemia). Diagnosis of BPPV and exclusion of other vestibular disorders were based on a comprehensive neurotological examination, including detailed history-taking, bedside vestibular tests (e.g., head impulse test, assessment for spontaneous and gaze-evoked nystagmus), and when clinically indicated, video nystagmography and vestibular evoked myogenic potentials to rule out conditions like vestibular neuritis or central lesions. All BPPV patients were enrolled during the acute symptomatic phase. Participants presented to the hospital within 24–48 h after the onset of severe vertigo episodes.

### Clinical and demographic data

Demographic variables (age, sex, weight, height) and comorbidities (hypertension, diabetes, and hyperlipidemia) were recorded. Body mass index (BMI) was computed as weight (kg) divided by height squared (m^2^). Baseline characteristics, including smoking and alcohol consumption history, were collected via structured interviews (21).

### Psychological and sleep assessments

Participants underwent a series of validated assessments to evaluate psychological status and sleep quality. The following instruments were administered by trained interviewers following standardized protocols.

#### HAMA

The HAMA is a clinician-rated scale assessing anxiety severity across 14 items, each scored 0–4 (20). Total scores (0–56) are categorized as: <7 (no anxiety), 7–13 (possible), 14–20 (mild), 21–28 (moderate), ≥29 (severe) (22, 23).

#### HAMD

The 17-item HAMD quantifies depressive symptom severity, with items rated on 3- or 5-point scales (total 0–52). Conventional cutoffs are: <7 (normal), 7–17 (mild), 18–24 (moderate), >24 (severe) (23, 24).

#### PSQI

The PSQI assesses sleep quality over 1 month via 19 self-rated items generating seven component scores (0–3 each) (25). These yield a global score (0–21; higher = worse sleep), with established cutoffs: 0–5 (good), 6–10 (fair), 11–15 (moderate), 16–21 (poor) (25).

### Dizziness handicap inventory (DHI)

The DHI is a 25-item self-reported questionnaire designed to quantify the self-perceived handicapping effects imposed by dizziness and unsteadiness (26). The instrument assesses disability across three domains: Functional (9 items), Emotional (9 items), and Physical (7 items). Each item is scored as 0 (no), 2 (sometimes), or 4 (yes). Subscale maximum scores are 36 (Functional/Emotional) and 28 (Physical), yielding a total score range of 0–100, with higher scores indicating greater perceived handicap (26). Total DHI scores can be categorized into three severity levels: mild handicap (0–30 points), moderate handicap (31–60 points), and severe handicap (61–100 points) (26). Patients with scores >60 demonstrate significantly greater functional impairment and increased fall risk compared to those with lower scores (26).

The Chinese version of the DHI has been previously validated in Chinese-speaking patients with dizziness, demonstrating good internal consistency (Cronbach’s *α* = 0.751–0.912) and test–retest reliability (*r* = 0.877–0.921) (27). While the DHI demonstrates sufficient reliability, evidence regarding its structural validity is inconsistent, with factor analyses failing to consistently support the original three-subscale structure (28). Current evidence suggests using only the total score rather than individual subscale scores (28). We acknowledge that self-reported DHI scores may be influenced by comorbid anxiety.

### Neurotransmitter measurement

Blood samples (5 mL) were collected into vacutainer serum separator tubes between 8:00–10:00 a.m. after an overnight fast and 30 min of supine rest. Sampling occurred within 48 h after the patient’s last vertigo episode. Serum was separated via centrifugation (3,000 rpm, 15 min, 4 °C) and stored at −80 °C. Catecholamines (epinephrine, norepinephrine, dopamine) were quantified using a YS EXACT9900MD high-performance liquid chromatography–tandem mass spectrometry (HPLC-MS/MS) system (Yingsheng Biotechnology, Shandong, China). Separation was achieved on a C18 column (2.1 × 100 mm, 1.8 μm) maintained at 40 °C. The mobile phase used was a gradient system composed of 0.1% formic acid in water and acetonitrile, flowing at a rate of 0.3 mL/min. In the mass spectrometry part, detection was carried out in positive electrospray ionization (ESI+) mode. Multiple reaction monitoring (MRM) was employed with optimized collision energies for each analyte: for epinephrine, the transition was m/z 184.1 → 166.1 with a collision energy of 15 eV; for norepinephrine, it was m/z 170.1 → 152.1 with a collision energy of 12 eV; and for dopamine, it was m/z 154.1 → 137.1 with a collision energy of 10 eV. All serum samples were analyzed in a single batch after complete participant enrollment to minimize inter-assay variability.

### Statistical analysis

Data were analyzed using SPSS 22.0 (IBM Corp., Armonk, NY, United States) and GraphPad Prism 8.0 (GraphPad Software, San Diego, CA, United States). Normality of continuous variables was assessed using the Shapiro–Wilk test. Continuous variables with normal distribution were expressed as mean ± standard deviation (SD) and compared via independent *t*-tests. Categorical variables were analyzed using *χ*^2^ tests. For correlation analyses, scatter plots were first inspected to assess the nature of the relationships between variables. As some associations appeared non-linear, Spearman’s rank correlation coefficient was employed to evaluate monotonic relationships between three biological indicators (dopamine, epinephrine, and norepinephrine) and clinical rating scales (DHI, Physical Subindex, Emotional Subindex, Functional Subindex, HAMA, HAMD, and PSQI). Statistical significance was set at *p* < 0.05.

## Results

### Baseline characteristics

This study enrolled 310 BPPV patients who met the inclusion criteria and 300 healthy controls. The demographic and clinical characteristics of the study population were summarized in Table 1. Among the 310 BPPV patients, the posterior semicircular canal was the most commonly affected (206 cases, 66.5%), followed by the horizontal canal (102 cases, 32.9%) and the anterior canal (2 cases, 0.6%). No patients presented with multi-canal involvement. Regarding laterality, the right ear was involved in 170 patients (54.8%), the left ear in 138 patients (44.5%), and bilateral involvement was observed in 2 patients (0.6%).

**Table 1:** Comparison of demographic and clinical characteristics between controls and BPPV patients.

Variable	BPPV group (*n* = 310)	Control group (*n* = 300)	Test statistic	*P*
Age (years)	57.33 ± 6.38	56.21 ± 6.09	*t* = 1.249	0.206
Male, *n* (%)	149 (48.1%)	167 (55.7%)	*χ*^2^ = 0.434	0.501
BMI (kg/m^2^)	23.58 ± 1.79	23.85 ± 1.76	*t* = 0.532	0.589
Hypertension, *n* (%)	81 (26.1%)	65 (21.7%)	*χ*^2^ = 0.356	0.230
Diabetes, *n* (%)	65 (21.0%)	66 (22.0%)	*χ*^2^ = 0.368	0.532
Smoking History, *n* (%)	84 (27.1%)	76 (25.3%)	*χ*^2^ = 0.323	0.567
Alcohol Consumption History, *n* (%)	152 (49.0%)	144 (48.0%)	*χ*^2^ = 0.400	0.522
Hyperlipidemia, *n* (%)	140 (45.2%)	52 (17.3%)	*χ*^2^ = 0.623	0.416
BPPV-specific characteristics				
Affected canal, *n* (%)				
Posterior canal	206 (66.5%)	–	–	–
Horizontal canal	102 (32.9%)	–	–	–
Anterior canal	2 (0.6%)	–	–	–
Multi-canal involvement	0 (0.0%)	–	–	–
Laterality, *n* (%)				
Left	138 (44.5%)	–	–	–
Right	170 (54.8%)	–	–	–
Bilateral	2 (0.6%)	–	–	–

Data for continuous variables are presented as mean ± standard deviation (SD). Categorical variables are presented as number (%). *P*-values were calculated using the independent *t*-test for continuous variables and the chi-square test for categorical variables. Statistical significance was set at *p* < 0.05. BPPV, benign paroxysmal positional vertigo; BMI, body mass index; SD, standard deviation.

No significant differences were observed between BPPV patients and controls in age (*p* = 0.206), sex (*p* = 0.501), BMI (*p* = 0.589), smoking history (*p* = 0.567), alcohol consumption history (*p* = 0.522), or comorbidities including hypertension (*p* = 0.230), diabetes (*p* = 0.532), and hyperlipidemia (*p* = 0.416). These non-significant differences confirmed the statistical comparability of the two groups for key demographic variables.

### Psychological and sleep outcomes

**Figure 1:**
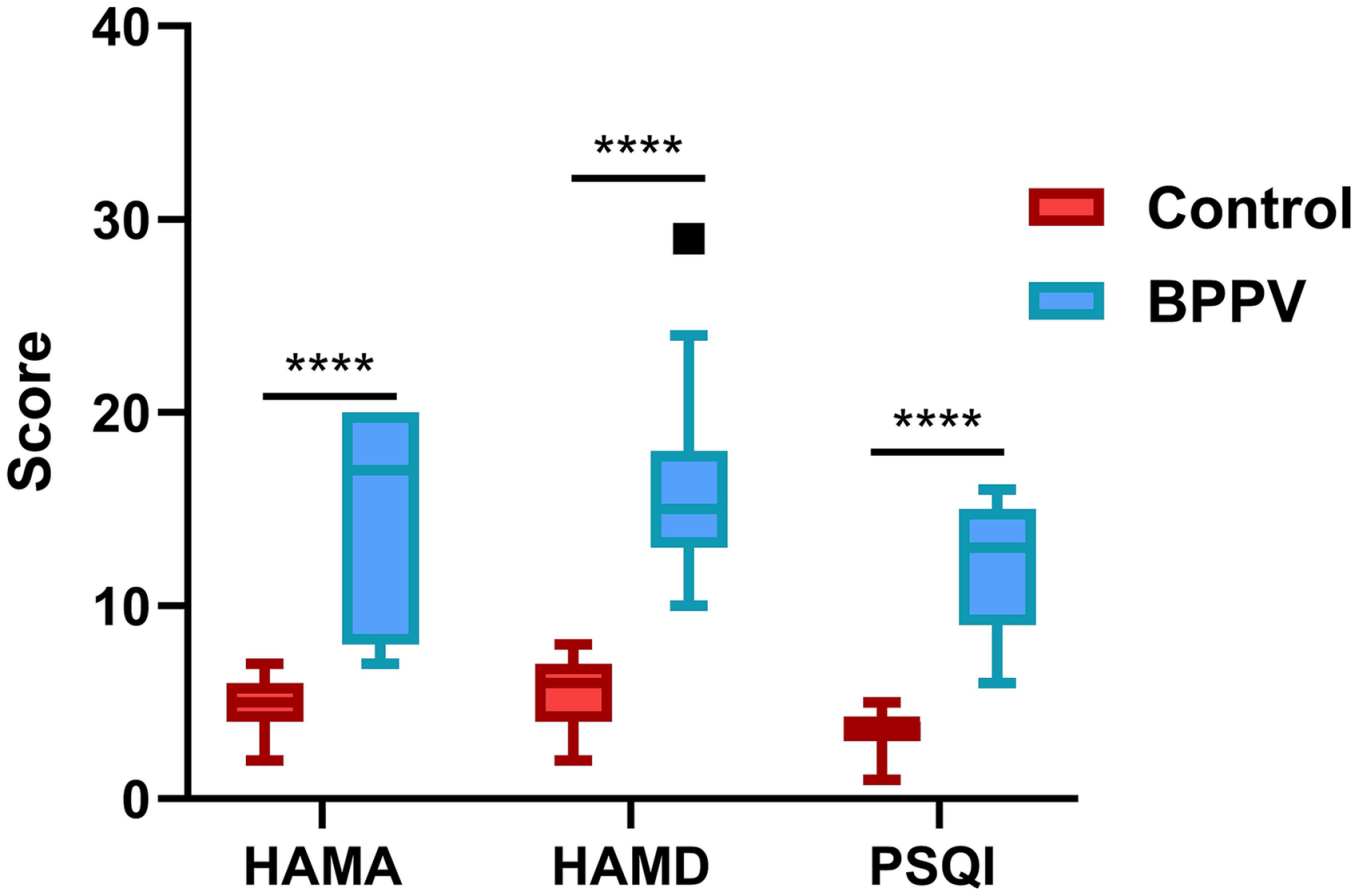
Boxplots showing the distribution of HAMA, HAMD, and PSQI scores in BPPV patients and healthy controls. The box represents the interquartile range (IQR), the horizontal line within the box indicates the median, and the whiskers extend to the most extreme data points within 1.5 × IQR; data points beyond this range are plotted as individual outliers. PSQI, Pittsburgh Sleep Quality Index; HAMA, Hamilton Anxiety Rating Scale; HAMD, Hamilton Depression Rating Scale. Statistical comparisons were performed using the independent *t*-test; ^****^p < 0.0001.

All participants underwent the assessments of HAMA, HAMD, and PSQI. Compared with the Control group, BPPV patients exhibited significantly higher HAMA (*p* < 0.0001), HAMD (*p* < 0.0001), and PSQI scores (*p* < 0.0001) (Table 2 and Figure 1).

**Table 2:** Comparison of psychological and sleep assessment scores between controls and BPPV patients.

Group	HAMA	HAMD	PSQI
Control	5.00 ± 1.34	5.55 ± 1.86	3.36 ± 1.12
BPPV	15.09 ± 5.32	16.18 ± 5.64	12.00 ± 3.52
*P*	<0.0001	<0.0001	<0.0001

Data are presented as mean ± standard deviation. *p*-values were calculated using the independent *t*-test. PSQI, Pittsburgh Sleep Quality Index; HAMA, Hamilton Anxiety Rating Scale; HAMD, Hamilton Depression Rating Scale.

### Neurotransmitter levels

**Figure 2:**
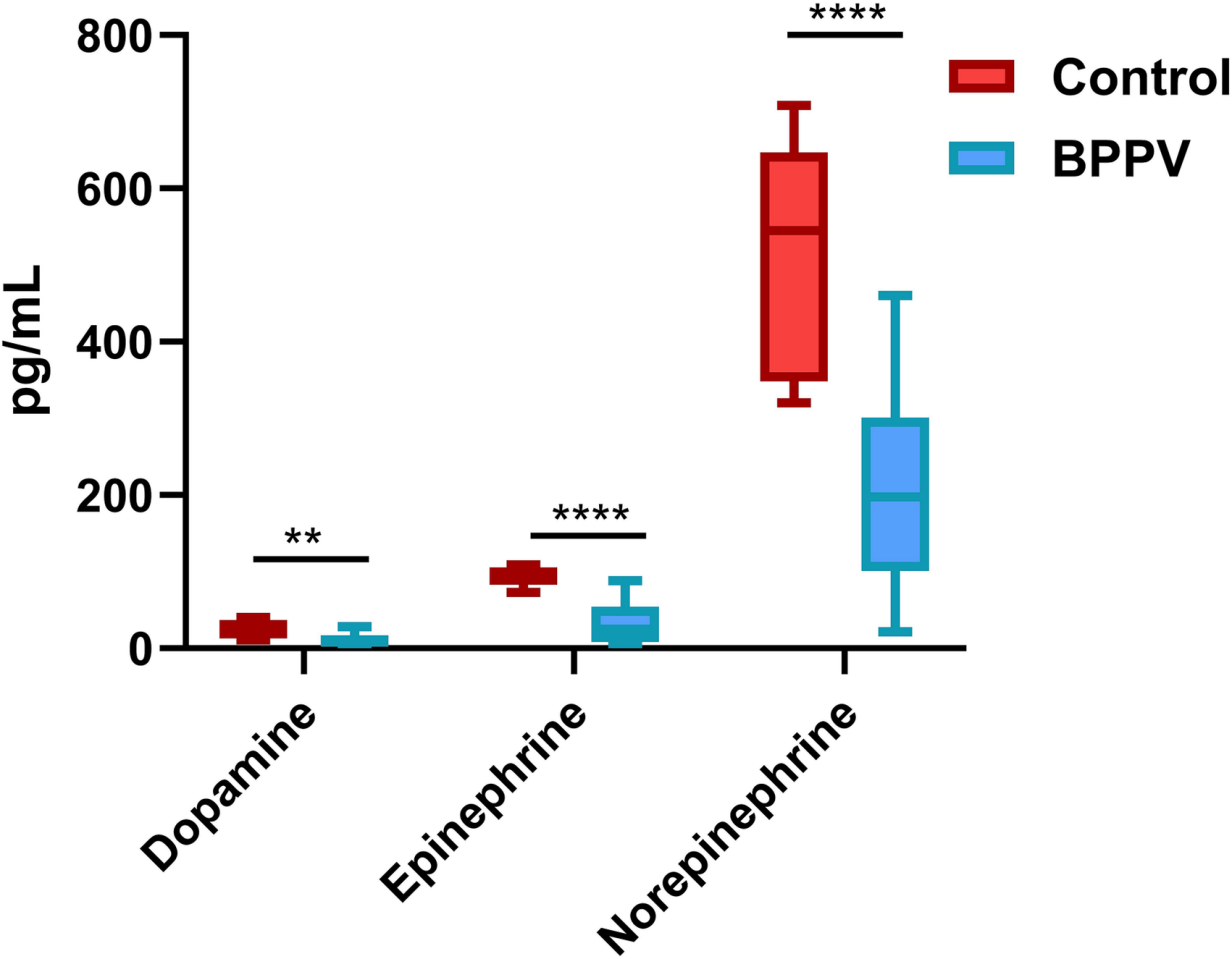
Boxplots showing the distribution of serum neurotransmitter concentrations (dopamine, epinephrine, and norepinephrine) in BPPV patients and healthy controls. The box represents the IQR, the horizontal line within the box indicates the median, and the whiskers extend to the most extreme data points within 1.5 × IQR. Statistical comparisons were performed using the independent *t*-test; ^****^p < 0.0001, ^**^p < 0.01.

The chromatographic separation of dopamine, epinephrine, and norepinephrine was achieved under optimized conditions (Supplementary Figures 1–3). Serum dopamine levels were significantly reduced in BPPV patients compared to controls (*p* = 0.0048). Similarly, relative to the Control group, the levels of epinephrine and norepinephrine in serum were significantly decreased (*p* < 0.0001) (Table 3 and Figure 2).

**Table 3:** Comparison of serum neurotransmitter concentrations between controls and BPPV patients.

Group	Dopamine (pg/mL)	Epinephrine (pg/mL)	Norepinephrine (pg/mL)
Control	25.85 ± 11.45	92.69 ± 12.14	500.58 ± 147.05
BPPV	11.71 ± 9.38	34.21 ± 27.40	210.12 ± 121.84
*P*	0.0048	<0.0001	<0.0001

### Correlation analysis

**Figure 3:**
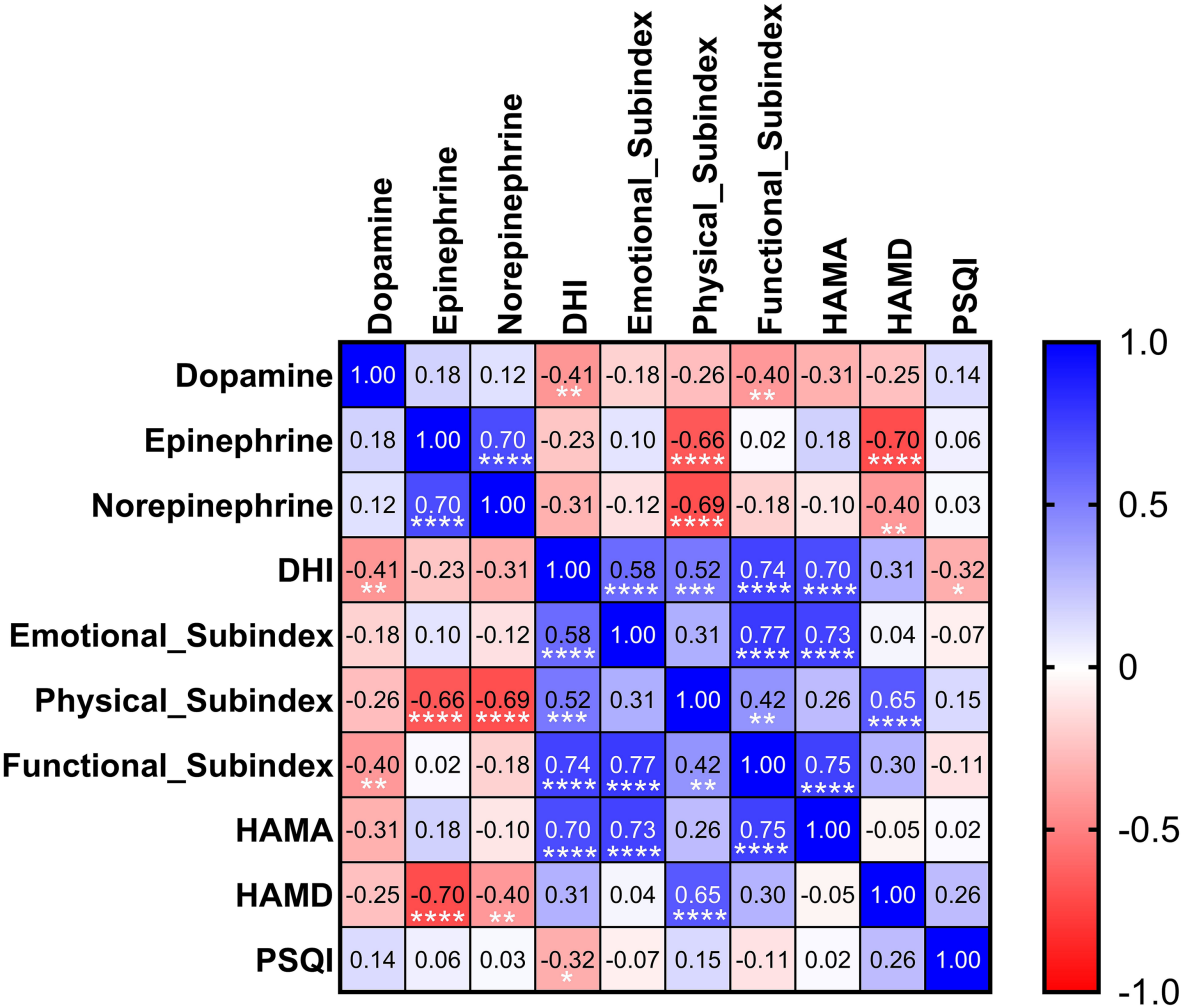
A heatmap of the Spearman correlation matrix to explore the relationships between three biological indicators (dopamine, epinephrine, and norepinephrine) and several scoring groups, with cool colors (e.g., blue, gray) indicating strong positive correlations and warm colors (e.g., red, orange) indicating strong negative correlations. ^****^p < 0.0001, ^***^p < 0.001, ^**^p < 0.01, ^*^p < 0.05.

In this study, we aimed to explore the relationships between three biological indicators (dopamine, epinephrine, and norepinephrine) and clinical rating scales (DHI, Physical Subindex, Emotional Subindex, Functional Subindex, HAMA, HAMD, and PSQI). We employed the Spearman correlation analysis method, as scatter plot inspection revealed non-linear associations between some variables. The Spearman correlation coefficient matrix was calculated and presented in Figure 3. Full correlation coefficients and *p*-values were provided in Supplementary Table 1.

For the biological indicators, a strong positive correlation was observed between epinephrine and norepinephrine levels (*r* = 0.699, *p* < 0.0001), indicating a coordinated regulatory relationship between these two catecholamines.

Regarding the relationships between biological indicators and scoring groups, serum epinephrine levels showed strong negative correlations with the Physical subindex of the DHI (*r* = −0.658, *p* < 0.0001) and with HAMD scores (*r* = −0.704, *p* < 0.0001), suggesting that lower epinephrine levels were associated with greater physical impairment and more severe depressive symptoms. Serum norepinephrine levels demonstrated a strong negative correlation with the Physical subindex of the DHI (*r* = −0.694, *p* < 0.0001). In addition, norepinephrine showed moderate negative correlations with HAMD scores (*r* = −0.404, *p* = 0.010). Serum dopamine levels exhibited moderate negative correlations with the Functional subindex of the DHI (*r* = −0.404, *p* = 0.010) and with the DHI total score (*r* = −0.412, *p* = 0.008).

Among the scoring groups, a remarkably strong positive correlation (*r* = 0.701, *p* < 0.0001) was found between DHI and HAMA, suggesting that these two indicators may be influenced by common factors and have similar implications in assessing certain physiological or psychological characteristics. The Emotional subindex correlated strongly with HAMA scores (*r* = 0.734, *p* < 0.0001). The Physical subindex showed a strong positive correlation with HAMD scores (*r* = 0.650, *p* < 0.0001). Additionally, the DHI total score showed a moderate negative correlation with PSQI (*r* = −0.325, *p* = 0.041), indicating that greater dizziness handicap was associated with poorer sleep quality.

## Discussion

Benign paroxysmal positional vertigo, a common vestibular disorder, is increasingly recognized for its association with psychological and neurobiological disturbances. While prior studies have highlighted the bidirectional link between BPPV and anxiety, the underlying molecular mechanisms, particularly involving neurotransmitter systems, remain poorly understood (29, 30). Our study addresses this gap by demonstrating significant reductions in serum dopamine, epinephrine, and norepinephrine in BPPV patients, alongside elevated anxiety, depression, and sleep dysfunction scores. These findings suggest a critical role for monoaminergic pathways in mediating the relationship between vestibular dysfunction and emotional/sleep disturbances.

This study revealed significantly lower serum levels of dopamine, epinephrine, and norepinephrine in BPPV patients compared to healthy controls, highlighting the critical role of monoaminergic pathways in vestibular dysfunction. Dopamine and norepinephrine modulate brainstem-vestibular nucleus interactions, regulating vestibular sensitivity and central compensation mechanisms (31). Norepinephrine enhances the gain of vestibular signals in the pontine reticular formation, and its deficiency may impair central adaptation to otolith dysfunction, exacerbating dizziness and postural instability (32, 33). The observed neurotransmitter deficits were closely linked to clinical measures of physical and emotional impairment. Specifically, serum epinephrine levels showed strong negative correlations with the Physical subindex of the DHI, indicating that lower epinephrine levels are associated with greater physical impairment. This finding aligns with the established role of epinephrine in the hypothalamic–pituitary–adrenal axis and suggests that epinephrine deficiency may contribute to the comorbid physical and affective disturbances observed in BPPV patients. Similarly, norepinephrine levels were strongly negatively correlated with the Physical subindex of the DHI, reinforcing the critical role of this neurotransmitter in modulating vestibular-related physical function. Consistent with this notion, Power et al. have reported that anxiety scores inversely correlated with norepinephrine recovery after canalith repositioning, suggesting that neurotransmitter normalization may alleviate psychological symptoms and functional impairment (34). Furthermore, dopamine levels showed moderate negative correlations with the Functional subindex of the DHI and the total DHI score, suggesting that dopaminergic deficits may contribute to functional disability in BPPV, potentially through its modulatory effects on motor planning and vestibular nucleus activity. In addition, the use of HPLC-MS/MS in this study offered a precise and sensitive method to quantify these neurotransmitters, surpassing traditional enzyme-linked immunosorbent assays in detecting subtle biochemical changes. This technique revealed a strong positive correlation between epinephrine and norepinephrine, suggesting a coordinated regulatory mechanism between these two catecholamines in vestibular pathways.

Benign paroxysmal positional vertigo patients exhibited significantly higher anxiety (HAMA) and depression (HAMD) scores than controls, with a striking positive correlation between DHI and HAMA, underscoring the interplay between physical symptoms and psychological distress. This aligns with Kozak et al. (35), who have found that 39.1% of BPPV patients had comorbid anxiety or depression, a prevalence far exceeding that in healthy populations. The bidirectional relationship likely operates through two pathways: recurrent vertigo triggers fear of falls and activity avoidance, directly increasing functional disability; conversely, anxiety amplifies vestibular sensitivity, creating a vicious cycle of heightened symptom perception (20, 36). Notably, this anxiety may persist even after successful canalith repositioning, as patients may develop conditioned fear of head movements that previously triggered their vertigo, even when such movements no longer provoke vestibular symptoms (37, 38). Such anticipatory anxiety can independently perpetuate functional disability and psychological distress, reinforcing the need for integrated interventions that address both the peripheral vestibular pathology and its central behavioral consequences. Our correlation analysis showed that lower epinephrine and norepinephrine levels were significantly associated with more severe depressive symptoms, suggesting neurotransmitter systems as key targets for integrated psychological and pharmacological interventions. Additionally, the Emotional subindex of the DHI was strongly correlated with HAMA scores, reinforcing the notion that emotional dysfunction in BPPV is closely intertwined with anxiety symptomatology. The interdependence between emotional state and functional disability underscores the importance of holistic management strategies that combine vestibular rehabilitation with psychological interventions to improve outcomes.

Sleep dysfunction, evident in elevated PSQI scores and reduced sleep efficiency in BPPV patients, reflects a reciprocal relationship with vestibular symptoms. Positional changes during sleep, a common trigger for vertigo, disrupt sleep architecture, particularly reducing deep sleep and increasing wakefulness after sleep onset (39). This aligns with research showing that BPPV patients experience significantly worse sleep quality than healthy controls, with poor sleep predicting higher recurrence rates (40). Although neurotransmitter imbalances did not directly correlate with PSQI scores in our study, the DHI total score showed a moderate negative correlation with PSQI, suggesting that greater dizziness handicap is associated with poorer sleep quality. Beyond neurochemical mechanisms, behavioral pathways may also contribute to sleep disturbances. Anticipatory anxiety, driven by fear of vertigo during sleep-position changes, may independently perpetuate sleep disruption, creating a behavioral feedback loop wherein psychological distress amplifies vestibular sensitivity. These findings highlight sleep quality as a critical modifiable factor in BPPV management, warranting interventions to improve sleep hygiene alongside the canalith repositioning procedure.

Several limitations of this study should be acknowledged. First, the cross-sectional design precludes any causal inferences regarding the observed associations between neurotransmitter levels, psychological symptoms, and sleep disturbances. Although we identified significant correlations, it remains unclear whether the neurotransmitter deficits contribute to the development of anxiety and depression, or whether the psychological distress associated with BPPV leads to alterations in monoamine levels. A bidirectional relationship is plausible, and longitudinal studies are needed to clarify the temporal and causal pathways. Second, the assessment of sleep quality relied solely on the PSQI, a subjective self-report measure. While the PSQI is a well-validated instrument, the absence of objective sleep assessments such as polysomnography limits our ability to characterize sleep architecture abnormalities or to distinguish between sleep disturbances directly related to vestibular dysfunction and those associated with comorbid psychiatric symptoms. Future studies incorporating objective sleep measurements would provide a more comprehensive understanding of the interplay between BPPV, sleep, and psychological health. Third, our investigation focused on three catecholamines, dopamine, epinephrine, and norepinephrine, while other important neurotransmitters, particularly serotonin, which plays a critical role in mood regulation and sleep–wake cycles, were not measured. The inclusion of serotonin and other relevant neurochemicals in future studies would allow for a more complete characterization of the neurobiological underpinnings of BPPV comorbidities. Fourth, the generalizability of our findings may be limited by the single-center design and the relatively homogeneous study population. Multicenter studies with diverse cohorts are warranted to validate our results.

This study advances our understanding of BPPV by linking neurochemical, psychological, and sleep-related disturbances, revealing a complex interplay that influences patient prognosis. The identification of serum neurotransmitters as potential biomarkers for psychological comorbidities provides a potential biomarker for identifying psychological comorbidities and a possible target for integrated therapeutic strategies. Clinically, these findings emphasize the need for comprehensive evaluations that include mental health and sleep quality assessments, particularly in patients with recurrent or refractory BPPV. Future research should explore the causal mechanisms underlying these associations and test the efficacy of combined interventions targeting neurotransmitter balance, anxiety, and sleep disorders.

## Data Availability

The original contributions presented in the study are included in the article/Supplementary material, further inquiries can be directed to the corresponding author.
