# Supplementary material for: Serum neurotransmitter imbalances in benign paroxysmal positional vertigo: correlations with anxiety, depression, and sleep quality
Source: Front Neurol. 2026 Apr 10;17:1798705. doi: 10.3389/fneur.2026.1798705 (PMC13106000; doi:10.3389/fneur.2026.1798705)

**Supplementary Figure 1**. Representative chromatograms of dopamine from the Control and BVVP groups.


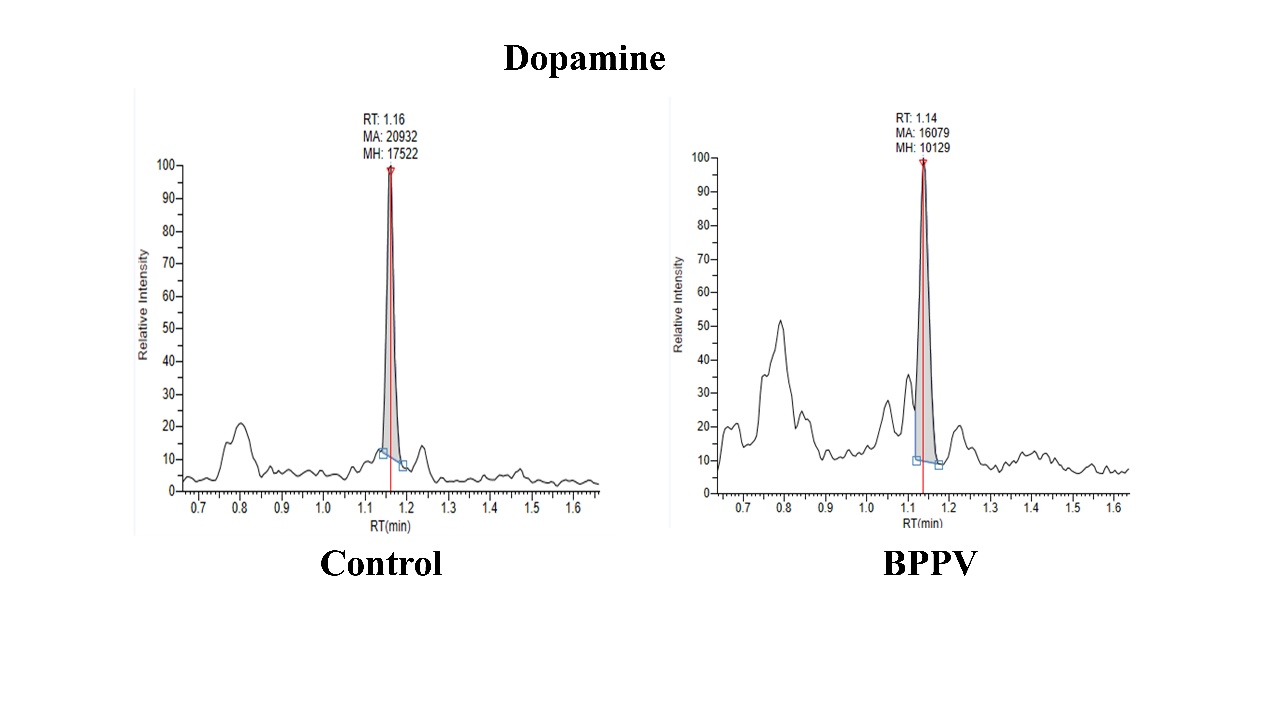


**Supplementary Figure 2**. Representative chromatograms of norepinephrine from the Control and BVVP groups.


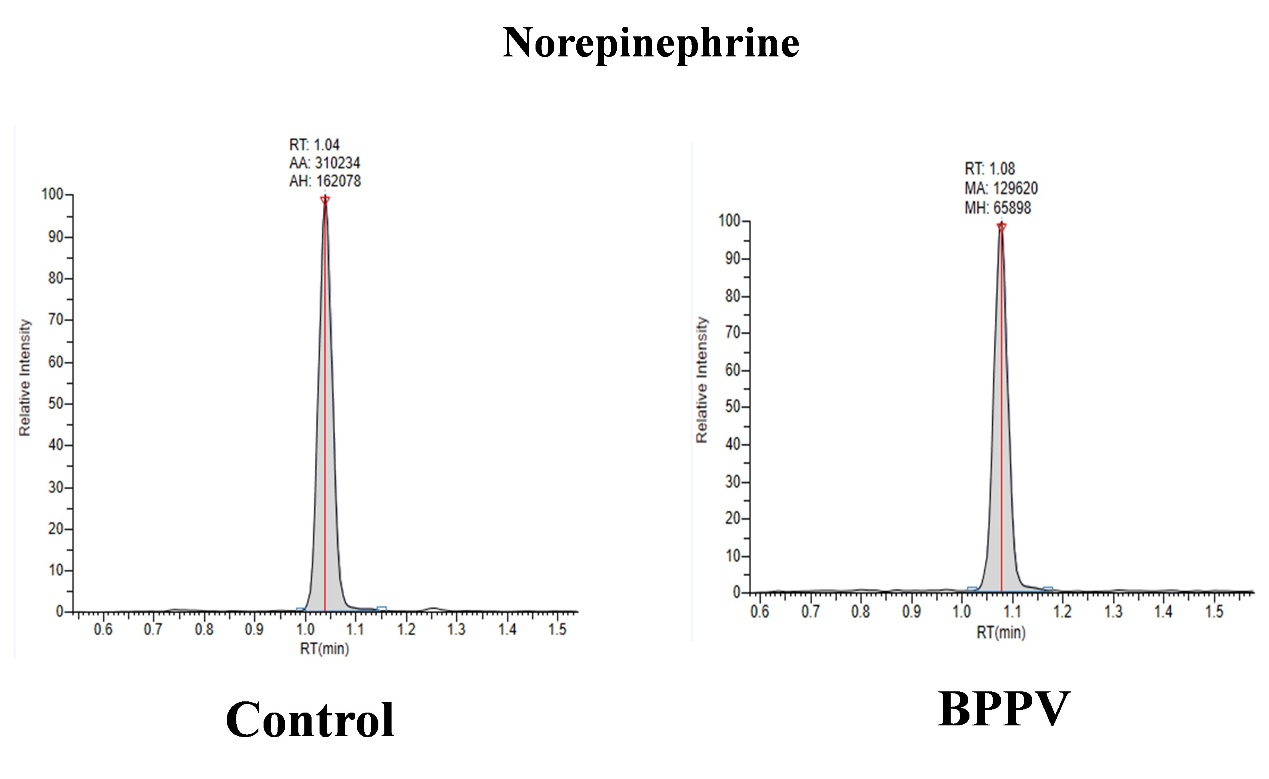


**Supplementary Figure 3**. Representative chromatograms of epinephrine from the Control and BVVP groups.


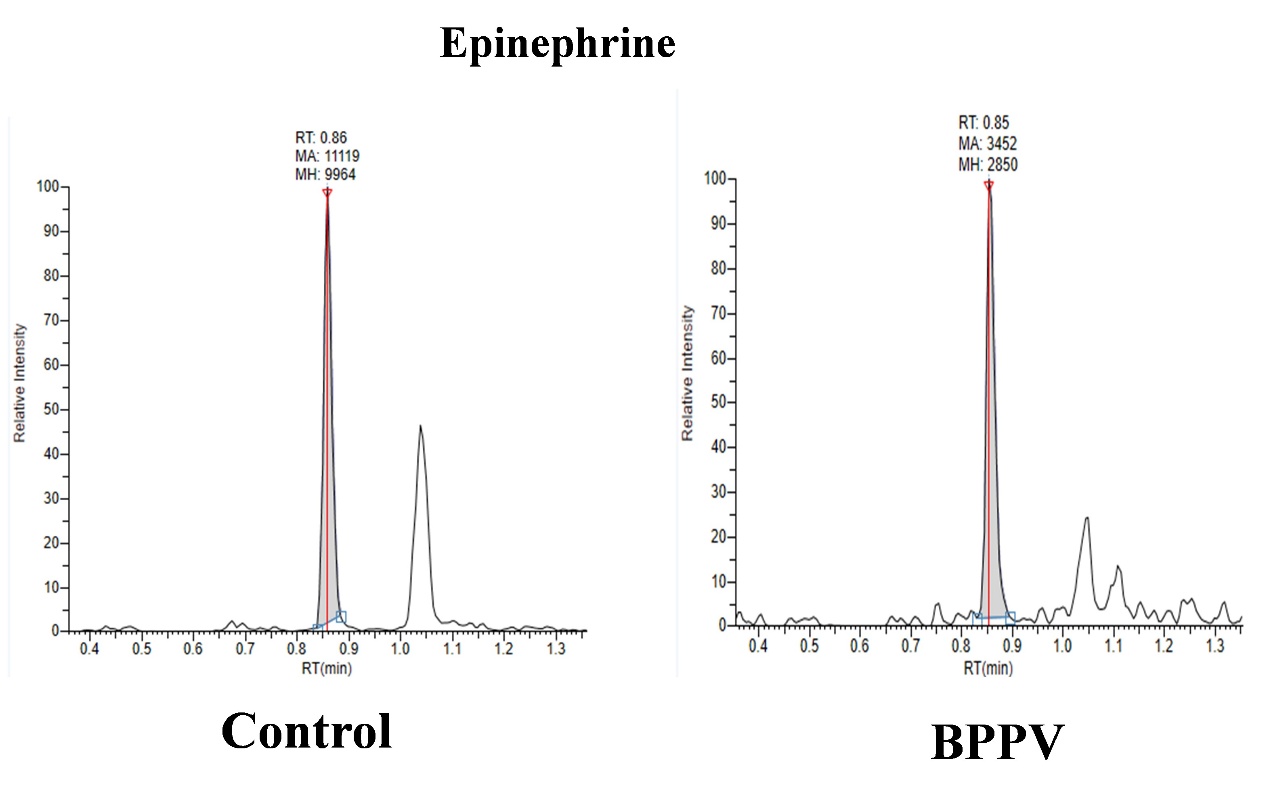

Supplement: Supplementary file 1 [file Supplementary_file_1.docx]
