# Supplementary material for: Serum neurotransmitter imbalances in benign paroxysmal positional vertigo: correlations with anxiety, depression, and sleep quality
Source: Front Neurol. 2026 Apr 10;17:1798705. doi: 10.3389/fneur.2026.1798705 (PMC13106000; doi:10.3389/fneur.2026.1798705)
Supplement: Supplementary file 2 [file Table_1.docx]

| Variable | Dopamine | Epinephrine | Norepinephrine | DHI | Emotional | Physical | Functional | HAMA | HAMD | PSQI |
| --- | --- | --- | --- | --- | --- | --- | --- | --- | --- | --- |
| ****Dopamine**** | 1 | 0.176 (0.279) | 0.120 (0.461) | ****-0.412 (0.008)**** | -0.183 (0.259) | -0.259 (0.106) | ****-0.404 (0.010)**** | -0.310 (0.052) | -0.251 (0.119) | 0.139 (0.393) |
| ****Epinephrine**** | 0.176 (0.279) | 1 | ****0.699 (<0.0001)**** | -0.227 (0.158) | 0.103 (0.528) | ****-0.658 (<0.0001)**** | 0.022 (0.894) | 0.179 (0.269) | ****-0.704 (<0.0001)**** | 0.063 (0.701) |
| ****Norepinephrine**** | 0.120 (0.461) | ****0.699 (<0.0001)**** | 1 | -0.307 (0.054) | -0.124 (0.447) | ****-0.694 (<0.0001)**** | -0.183 (0.259) | -0.099 (0.541) | ****-0.404 (0.010)**** | 0.026 (0.872) |
| ****DHI**** | ****-0.412 (0.008)**** | -0.227 (0.158) | -0.307 (0.054) | 1 | ****0.577 (<0.0001)**** | ****0.523 (<0.001)**** | ****0.741 (<0.0001)**** | ****0.701 (<0.0001)**** | 0.306 (0.055) | ****-0.325 (0.041)**** |
| ****Emotional**** | -0.183 (0.259) | 0.103 (0.528) | -0.124 (0.447) | ****0.577 (<0.0001)**** | 1 | 0.310 (0.051) | ****0.772 (<0.0001)**** | ****0.734 (<0.0001)**** | 0.036 (0.826) | -0.065 (0.690) |
| ****Physical**** | -0.259 (0.106) | ****-0.658 (<0.0001)**** | ****-0.694 (<0.0001)**** | ****0.523 (<0.001)**** | 0.310 (0.051) | 1 | ****0.417 (0.007)**** | 0.261 (0.104) | ****0.650 (<0.0001)**** | 0.146 (0.368) |
| ****Functional**** | ****-0.404 (0.010)**** | 0.022 (0.894) | -0.183 (0.259) | ****0.741 (<0.0001)**** | ****0.772 (<0.0001)**** | ****0.417 (0.007)**** | 1 | ****0.746 (<0.0001)**** | 0.297 (0.063) | -0.111 (0.496) |
| ****HAMA**** | -0.310 (0.052) | 0.179 (0.269) | -0.099 (0.541) | ****0.701 (<0.0001)**** | ****0.734 (<0.0001)**** | 0.261 (0.104) | ****0.746 (<0.0001)**** | 1 | -0.046 (0.779) | 0.023 (0.889) |
| ****HAMD**** | -0.251 (0.119) | ****-0.704 (<0.0001)**** | ****-0.404 (0.010)**** | 0.306 (0.055) | 0.036 (0.826) | ****0.650 (<0.0001)**** | 0.297 (0.063) | -0.046 (0.779) | 1 | 0.259 (0.106) |
| ****PSQI**** | 0.139 (0.393) | 0.063 (0.701) | 0.026 (0.872) | ****-0.325 (0.041)**** | -0.065 (0.690) | 0.146 (0.368) | -0.111 (0.496) | 0.023 (0.889) | 0.259 (0.106) | 1 |

**Supplementary Table 1.** Spearman correlation matrix between serum neurotransmitter levels (dopamine, epinephrine, norepinephrine) and clinical assessment scores (DHI total and subscales, HAMA, HAMD, PSQI) in BPPV patients. Data are presented as correlation coefficients (r) with the corresponding P-values in parentheses.
